# Exploratory Characterization of Phenolic Compounds with Demonstrated Anti-Diabetic Activity in Guava Leaves at Different Oxidation States

**DOI:** 10.3390/ijms17050699

**Published:** 2016-05-11

**Authors:** Elixabet Díaz-de-Cerio, Vito Verardo, Ana María Gómez-Caravaca, Alberto Fernández-Gutiérrez, Antonio Segura-Carretero

**Affiliations:** 1 Department of Analytical Chemistry, Faculty of Sciences, University of Granada, Avd. Fuentenueva s/n, 18071 Granada, Spain; ediazdecerio002@correo.ugr.es (E.D.-d.-C.); anagomez@ugr.es (A.M.G.-C.); albertof@ugr.es (A.F.-G.); 2 Functional Food Research and Development Center, Health Science Technological Park, Avd. del Conocimiento, Bioregion building, 18100 Granada, Spain; 3 Department of Chemistry and Physics (Analytical Chemistry Area) and Research Centre for Agricultural and Food Biotechnology (BITAL), Agrifood Campus of International Excellence, ceiA3, University of Almería, Carretera de Sacramento s/n, E-04120 Almería, Spain

**Keywords:** *Psidium guajava* L., HPLC-DAD-ESI-QTOF-MS, phenolic compounds, gallic and ellagic derivatives, flavonols, cyanidin-glucoside

## Abstract

*Psidium guajava* L. is widely used like food and in folk medicine all around the world. Many studies have demonstrated that guava leaves have anti-hyperglycemic and anti-hyperlipidemic activities, among others, and that these activities belong mainly to phenolic compounds, although it is known that phenolic composition in guava tree varies throughout seasonal changes. Andalusia is one of the regions in Europe where guava is grown, thus, the aim of this work was to study the phenolic compounds present in Andalusian guava leaves at different oxidation states (low, medium, and high). The phenolic compounds in guava leaves were determined by HPLC-DAD-ESI-QTOF-MS. The results obtained by chromatographic analysis reported that guava leaves with low degree of oxidation had a higher content of flavonols, gallic, and ellagic derivatives compared to the other two guava leaf samples. Contrary, high oxidation state guava leaves reported the highest content of cyanidin-glucoside that was 2.6 and 15 times higher than guava leaves with medium and low oxidation state, respectively. The QTOF platform permitted the determination of several phenolic compounds with anti-diabetic properties and provided new information about guava leaf phenolic composition that could be useful for nutraceutical production.

## 1. Introduction

*Psidium guajava* (*P. guajava*) L., from the *Myrtaceae* family, is common throughout tropical and subtropical areas [1] and Andalusia is one of the regions in Europe where guava is grown. Moreover, it is widely used like food and in folk medicine all around the world. Many studies have demonstrated that guava leaves have anti-hyperglycemic and anti-hyperlipidemic activities [2,3,4], among others, and these biological activities have mainly been related to the phenolic compounds [5].

Nowadays, alternative therapeutic strategies based on the use of phenolic compounds in food products as “functional foods” and “nutraceuticals” are being developed. In fact, the capacity of plant-derived foods to reduce the risk of chronic diseases has been demonstrated [6].

It is known that *P. guajava* L. shows different phenological stages throughout its vegetative period in response to environmental conditions [7], because of that, it has been seen that the accumulation of specific compounds such as anthocyanins changes [8]. Furthermore, the response of the different classes of phenolic compounds, especially flavonoids, also vary substantially [9]. This fact plays an important role in finding the best conditions of the leaf in order to obtain the best recovery of the target compounds for the development of a promising alternative source for ameliorating diabetes complications [2,3,4].

In this sense, spectrophotometric analyses are still helpful for a preliminary identification and quantification [10]; however, LC-MS has opened up new approaches for the structural characterization of target compounds. Moreover, LC-TOF-MS can provide tentative identification of unknown peaks, due to accurate-mass measurement [11]. So, both UV-VIS diode array and mass spectrometry coupled to HPLC have been proved as most appropriate analytical techniques for phenolic compounds in many matrices [10,11]. Concerning guava leaves, most of the literature shows that quantification of the different classes of phenolic compounds is generally done via spectrophotometric analysis [12,13,14,15], although different analytical techniques, such as LC-DAD and LC-DAD-MS, are used to characterize the bioactive compounds present in guava leaves [3,4,16,17].

Despite these facts and to our knowledge, there is no literature taking into account the change that climatic conditions cause in phenolic composition and this information would be useful to choose the best raw material for nutraceutical scopes. Thus, the aim of this work was to study the phenolic compounds present in Andalusian guava leaves at different oxidation states (low, medium, and high) by HPLC-DAD-ESI-QTOF-MS.

## 2. Results and Discussion

### 2.1. Characterization of Phenolic Compounds

The HPLC-DAD-ESI-QTOF-MS analyses in negative mode permitted the identification and quantification of seventy-three phenolic compounds in guava leaves [18]. The individual compounds were quantified on the basis of their peak area and compared with calibration curves obtained with the corresponding standards and then expressed as μg/g of leaf dry weight (*d.w.*). Moreover, quantification of compounds for which no commercial standards were available, was achieved comparing with standard compounds bearing similar structures (Table 1).

**Figure 1:**
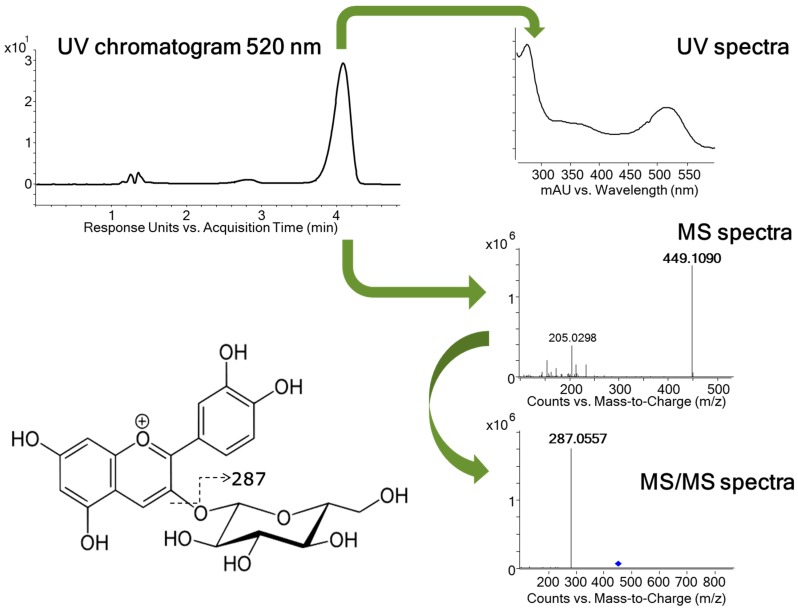
Fragmentation pattern of cyanidin-3-*O*-glucoside. MS/MS spectra has been obtained by auto MS/MS fragmentation.

Furthermore, the analysis in positive mode allowed the identification of an anthocyanin compound. The compound, with *m/z* 449.1090 presented its maximum of absorption at 280, 350, and 520 nm on the UV spectrum. The MS/MS analysis produced a fragment ion at 287 *m/z* corresponding to the loss of hexose unit (Figure 1). Due to the UV and MS spectrum and by co-elution with a commercial standard, this component has been identified as cyanidin-3-*O*-glucoside. To our knowledge, this compound has been identified for the first time in guava leaves. Limits of detection (LOD) and quantification (LOQ) calculated for the cyanidin-3-*O*-β-galactopyranoside standard were 0.007 and 0.024 mg/L.

In terms of concentration of the individual compounds (Table 1), in negative mode, leaves with lower oxidation state exhibited the highest amounts for almost all compounds quantified, followed by moderate oxidized leaves and, finally, highly oxidized leaves. Concentrations of several compounds tentatively identified [18], such as procyanidin tetramer and pentamer, galloyl-(epi)catechin trimer isomers 1 and 2, and quercetin glucuronide were found to be lower than the quantification limit for all samples. In contrast, and as it was expected due to the red coloration of the leaves at high oxidative state, in positive mode, opposite results were found (Table 1); the concentration of cyanidin-3-*O*-glucoside increased as the oxidation state of the leaves increased. At low oxidative state, the concentration of cyanidin-3-*O*-glucoside was 29.5 ± 0.2 μg/g leaf *d.w.* and it raised until 441.28 ± 0.04 μg/g leaf *d.w.* for the highest oxidation state.

**Figure 2:**
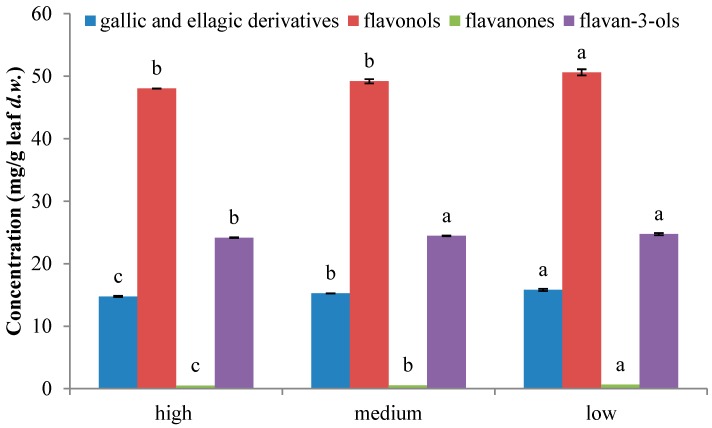
Quantification of different families of phenolic compounds present in guava leaves at different oxidative states. The different letter (a,b,c) in the same phenolic class means a significant difference (*p* ≤ 0.05).

Several works reported the quantification of some compounds such as ellagic acid, quercetin, gallic acid, catechin, and gallocatechin by HPLC-DAD in guava leaves [3,9,12,19]. Comparing the results, compounds in dried leaves are found in lower quantities than in the extract [3,12], although values in the same order of magnitude are noticed for quercetin [9] and greater amounts of catechin and gallocatechin are found comparing values with those obtained for dried leaves from Korea [19]. Furthermore, Zhu and coworkers [20] also isolated the major compounds in Chinese guava leaves: hyperoside, isoquercitrin, reynoutrin, guajaverin, avicularin, and also 2,4,6-trihydroxy-3,5-dimethylbenzophenone 4-*O*-(6″-*O*-galloyl)-β-d-glucopyranoside. Concentrations of reynoutrin, guajaverin, and avicularin isomers followed the same order as in the present leaves (reynoutrin < guajaverin < avicularin) and the opposite order was observed for the other isomers (isoquercitrin < hyperin). The compound 2,4,6-trihydroxy-3,5-dimethylbenzophenone 4-*O*-(6″-*O*-galloyl)-β-d-glucopyranoside was not detected in the present samples, in contrast, 2,6-dihydroxy-3-methyl-4-*O*-(6″-*O*-galloyl-β-d-glucopyranosyl)-benzophenone could be found. Differences noticed between these works could be because phenolic composition vary substantially among genotypes, seasons changes, ages, and damaged leaves, and location sites [7]. Regarding the different families present in leaves, extracts reported significant differences (*p* < 0.05), the lowest content of the different classes of phenolic compounds were found in leaves at the highest oxidation state, whereas the highest content was found in leaves at the lowest oxidation state (Figure 2). The major class of phenolic compounds in guava leaves samples was flavonols that ranged between 48.1 and 50.6 mg/g leaf *d.w.* The second class of polar compounds was represented by flavan-3-ols (24.2–24.7 mg/g leaf *d.w.*), followed by gallic and ellagic acid derivatives (14.8–15.8 mg/g leaf *d.w.*) and finally, by flavanones, that varied from 0.49 to 0.63 mg/g leaf *d.w.* Indeed, the contribution of reynoutrin, guajaverin, and avicularin isomers to total phenolic content (TPC) was predominant in this work, corresponding, on average, to 25% and 26% of the TPC, followed by hyperin and isoquercitrin, which supposed about 15%. Furthermore, myricetin derivatives contributed on 5% to TPC, morin and quercetin account between 3% and 5% of TPC from the different samples. Moreover, catechin was ranged between 7% and 10%, gallocatechin isomers varied between 7% and 8% of the total amount, and procyanidin B isomers represented a 6%.

The variance in concentration of the individual compounds (Table 1), and the differences between the families (Figure 2) is probably resulting from the different synthesis of secondary metabolites as response to the oxidative state of the leaves [9] and it could be explained by the formation of the compounds in the flavonoid pathway [21,22]. Most of the compounds present in guava leaves derivate from dihydroquercetin, precursor of both anthocyanins and flavonols. In fact, it has been noticed that leaves with low oxidation state presented greater amounts of flavonols, such as quercetin and myricetin derivatives, whereas the contents of the flavan-3ols catechin and gallocatechin, and the content of cyanidin-3-*O*-glucoside were lower. In contrast, the highest oxidative state showed lower amounts of quercetin and myricetin derivatives than low oxidation state, and higher concentration of catechin, gallocatechin, and cyanidin-3-*O*-glucoside than low oxidized leaves (Table 1). Chang *et al.* [16] also found for guava budding leaf tea that the presence of quercetin and its glycosides was larger than catechins and myricetin derivatives. Moreover, the increasing concentration of cyanidin-3-*O*-glucoside explained the dramatic red coloration of the leaves at higher oxidative states [21,22,23].

*P. guajava* leaves have traditionally been used in many countries to manage, control, and treat the diabetes [24], and its potential against diabetes mellitus type 2 has also been demonstrated in several works by the different parts of the plant, such as fruit, peel, pulp, seeds, and stem bark [25,26,27,28]. Singh *et al.* [29] reported that flavonoids are one of the major chemical constituents of plant species used in the management of diabetic complications. In fact, the anti-diabetic activity has mainly been attributed to a synergistic effect of the phenolic compounds present in the leaves [30]. This effect has been observed in half ripen guava fruit for *in vitro* and *in vivo* assays [25]. Authors reported that the effect is due to the total phenolic and flavonoid content in the fruit that was 40.13 ± 2.12 and 18.43 ± 1.22 mg/g of dry weight sample, respectively. Moreover, the recovery of total phenolic compounds in the peel was 58.7 ± 4.0 g gallic acid equivalent (GAE)/kg dry matter [31]. Ribeiro da Silva and coworkers [32] reported that the phenolic contents in the pulp was 1723.06 ± 111.58 mg GAE/100 g dry basis; in the seeds varied between 14.54 and 91.05 mg total phenols (TP)/100 g of defatted ground seeds in several solvents [33]; and the TPC of the stem bark, determined by Folin–Ciocalteu, was 1.15 ± 0.12 g GAE/100 g dry weight [34]. These values are lower than the ones summarized in the present work, so it could be supposed that guava leaves could be better anti-diabetic agents than the other parts of the plant.

Comparing the phenolic content of guava leaves to other plant leaves, different evidence has been found. The ethanol extract from *Telfairia occidentalis* leaves has also exhibited anti-hyperglycemic activity and has demonstrated strong inhibition of α-glucosidase and mild inhibition of α-amylase [35]. Authors related its activity to the phenolic and flavonoid content; however, its content was less than three order of magnitude than that reported in the present work. Additionally, other plants such as *Teucrium polium*, cinnamon and garlic, have generally presented lower phenolic contents than guava leaves [36,37,38] are also widely used in folk medicine for the treatment of diabetes [25]. In fact, different extracts from *Teucrium polium* (leaves, flowers, and stems) reported a TPC between 14.6 to 157.8 mg of GAE/g of extract [36], different parts of *Cinnamomum cassia* (barks, buds, and leaves) exhibited values for TPC from 6.3 to 9.5 g/100 g *d.w.* [37], and 3.4–10.8 mg GAE/g *d.w.* was the range of TPC found for different cultivars of garlic [38]. Additionally, several individual compounds isolated from different sources that have also been found in guava leaves have demonstrated anti-diabetic properties (Table 2). The principal activities related to these compounds are the inhibition of carbohydrate-hydrolysing enzymes due to the presence of myrciaphenone B [39], casuarictin and tellimagrandin I [40], flavonol glycosides (hyperin, isoquercitrin, reynoutrin, guajaverin, avicularin) [30,41], geraniin and catechin [42], quercetin [30], and cyanidin-3-*O*-β-glucoside [43]. Insulinomimetic activity has been attached to casuarinin, casuariin [40], procyanidin oligomers [44], and pedunculagin [45]. Moreover, geraniin, vescalagin, gallic acid, naringenin, morin, quercetin, catechin, epicatechin, and procyanidin B2 [42] exhibited anti-glycation activity, due to the inhibition of the formation of Amadori products and advanced glycation end-products (AGEs). At last, the improvement of postprandial hyperglycemia has been related to catechin and gallocatechin, among others [46].

### 2.2. Antioxidant Capacity and Total Phenolic Content

The evaluation of the antioxidant capacity is usually done comparing different methods in order to take into account the large number of factors that can influence the antioxidant action [47]. The choice of the two methods used in this work were assessed based on their different mechanisms: Trolox Equivalent Antioxidant Capacity (TEAC) assay estimates the ability to scavenge 2,2′-azinobis (3-ethylbenzothiazoline-6-sulfonate) (ABTS^•+^) radicals and ferric reducing capacity is evaluated by the Ferric Reducing Antioxidant Power (FRAP) method. As is shown in Table 3, significant differences have been found among the different oxidation states (*p* < 0.05), the lowest oxidation states reported the highest values for TEAC and FRAP (3.1 ± 0.1 mM eq Trolox/mg leaf *d.w.* and 5.4 ± 0.1 mM FeSO_4_/mg leaf *d.w.*, respectively) and decrease as the oxidation state increases. Comparing the results with those reported by Tachakittirungrod and coworkers [48] for guava leaves, similar values were accomplished for TEAC and higher values for FRAP. This can be due to the fact that phenolic compounds in Spanish leaves are more involved in the mechanism of reduction of oxidized intermediates in the chain reaction.

Moreover, high correlation was found between the antioxidant assays (*r* = 0.9978 and *p* < 0.001), in concordance with the data obtained for guava leaves [49], fruits [50], and in 30 plant extracts of industrial interest [51]. However, TEAC value seems to be higher than FRAP value for guava leaves [12,14,48].

Quantification of total phenolic compounds by HPLC-DAD-ESI-QTOF-MS revealed that the three extracts showed significant differences (*p* < 0.05) as is displayed in Table 3. The lowest oxidation state provided the highest content of total phenolic compounds (103 ± 2 mg/g leaf *d.w.*), followed by the medium and the highest oxidation state (92.0 ± 0.4 and 87.91 ± 0.04 mg/g leaf *d.w.*, respectively). The values obtained were lower than those reported by several authors that employed Folin–Ciocalteu method to quantify total phenolic compounds [12,14]. Even though the variance noticed is not great, it could be because the determination by HPLC presented only 50–60 percentage of total phenolic content [12]. Additionally, high correlation among TPC by HPLC, FRAP, and TEAC assays were found in the present work. In fact, positive correlation with *r* = 0.9921 (*p* < 0.001) was noticed between TPC by HPLC and FRAP and *r* = 0.9867 (*p* < 0.001) for TPC by HPLC and TEAC. Good correlations were also found between TPC by Folin–Ciocalteu and antioxidant activity was also noticed for guava leaves in literature [12,14,48].

## 3. Materials and Methods

### 3.1. Plant Material and Sample Preparation

Fresh guava leaves were harvested in Motril, Spain (36°44′43″N 3°31′14″W). The leaves were collected in February 2014 at different oxidation states (low, medium, and high) based on the difference of leaf color according to Hao [8]. The samples were air-dried at room temperature, ground, and extracted with ethanol:water 80/20 (*v*/*v*) by ultrasonics as was previously reported by Díaz-de-Cerio *et al.* [18].

### 3.2. Antioxidant Capacity Analysis

Trolox Equivalent Antioxidant Capacity (TEAC) and Ferric Reducing Antioxidant Power (FRAP) analysis were used to measure the antioxidant capacity.

For TEAC, ABTS radical cation was generated by reacting ABTS stock solution with 2.45 mM potassium persulfate in the dark at room temperature for 12–24 h before use. A calibration curve was prepared with different concentrations of Trolox (0–20 μM). The absorbance of ABTS radical cation was adjusted to 0.70 (±0.02) at 734 nm, and its change was measured [52].

To evaluate the reducing power, FRAP reagent (containing 2,4,6-tripyridyl-*S*-triazine (TPTZ), FeCl_3_ and acetate buffer) was prepared. An aqueous solution of Fe (II) was used for calibration (12.5–200 μM). The reduction was measured at 593 nm [53].

Results are expressed as mM eq Trolox/mg leaf *d.w.* and mM FeSO_4_/mg leaf *d.w.*, respectively.

### 3.3. HPLC-DAD-ESI-QTOF-MS Analysis

Chromatographic analyses were performed using an HPLC Agilent 1260 series (Agilent Technologies, Santa Clara, CA, USA) equipped with a binary pump, an online degasser, an autosampler, a thermostatically-controlled column compartment, and a UV-VIS diode array detector (DAD). The column was maintained at 25 °C. Phenolic compounds from *P. guajava* L. leaves were separated using a method previously reported by Gómez-Caravaca *et al.* [54], in positive mode, slightly modified. Briefly, a fused-core Poroshell 120, SB-C18 (3.0 mm × 100 mm, 2.7 μm) from Agilent Technologies (Agilent Technologies, Palo Alto, CA, USA) was used. The mobile phases were water plus 1% acetic acid (A) and acetonitrile (B). A multi-step linear gradient was applied as follows; 0 min, 5% B; 2 min, 7% B; 4 min, 9% B; 6 min, 12% B; 8 min, 15% B; 9 min, 16% B; 10 min, 17% B; 11 min, 17.5% B; 12 min, 18% B; 13 min, 100% B; 17 min, 100% B; 18 min, 5% B. The initial conditions were maintained for 5 min. The sample volume injected was 3 μL and the flow rate used was 0.8 mL·min^−1^.

MS analyses were carried out using a 6540 Agilent Ultra-High-Definition Accurate-Mass Q-TOF-MS coupled to the HPLC, equipped with an Agilent Dual Jet Stream electrospray ionization (Dual AJS ESI) interface, at the following conditions: drying gas flow (N_2_), 12.0 L/min; nebulizer pressure, 50 psi; gas drying temperature, 370 °C; capillary voltage, 3500 V; fragmentor voltage and scan range were 3500 V and *m*/*z* 50–1700. In positive mode, auto MS/MS experiments were carried out using the followings collision energy values: *m*/*z* 100, 40 eV; *m*/*z* 500, 45 eV; *m*/*z* 1000, 50 eV; and *m*/*z* 1500, 55 eV.

Additionally, phenolic compounds were also analyzed in negative mode using the chromatographic and the detection method described by Díaz-de-Cerio *et al.* [18].

Standard calibration curves for cyanidin-3-*O*-β-galactopyranoside, gallic acid, catechin, ellagic acid, naringenin, and rutin were prepared in the range of concentrations from the limit of quantification (LOQ) to 50 mg/L and five calibration points for each standard were run in triplicate (*n* = 3).

Integration and data elaboration were performed using MassHunter Workstation software (Agilent Technologies).

### 3.4. Statistical Analysis

The results reported in this study are the averages of three repetitions (*n* = 3). Fisher’s least significance difference (LSD) test and Pearson’s linear correlations, both at *p* < 0.05, were evaluated using Statistica 6.0 (2001, StatSoft, Tulsa, OK, USA).

## 4. Conclusions

HPLC coupled to QTOF-MS detector, which provides a molecular formula and the MS/MS data, permitted the analysis of the major phenolic compounds of guava leaves. The method performed in negative mode has proven to be successful in determining 73 compounds in the different guava leaves. Moreover, in positive mode, the analysis with QTOF analyzer and the co-elution with a standard solution allowed the identification of the cyanidin-glucoside. To our knowledge the cyanidin-glucoside, was identified for the first time in guava leaves. Quantification data, in negative mode, reported that leaves with low oxidation state presented the highest concentration of these compounds and decreased when the oxidation state raise. On the contrary, the state of oxidation affected significantly the cyanidin content. In fact, highest amount was detected in the leaves with high oxidation state.

Guava leaves seem to be a good source of phenolic compounds with described anti-diabetic properties since several compounds present in the leaves have been related for ameliorating the effects of diabetes mellitus disease, although this content varies due to the oxidative state of the leaf, so further studies should be carried out in order to evaluate the influence of the phenolic composition on the bioactivity of the extract.

## Figures and Tables

**Table 1:** Quantification (mean ± standard deviation (SD), *n* = 3) by HPLC-DAD-ESI-QTOF-MS in negative and positive mode of individual compound tentatively identified in *P. guajava* leaves for the different oxidative states.

No.	Compound	High	Medium	Low
*Negative mode*		Concentration (μg compound/g leaf *d.w.*)		
1	HHDP glucose Isomer	526 ± 2 ^c^	651 ± 19 ^b^	936 ± 10 ^a^
2	HHDP glucose Isomer	505 ± 3 ^c^	645 ± 3 ^b^	823 ± 16 ^a^
3	HHDP glucose Isomer	510 ± 11 ^c^	645 ± 20 ^b^	934 ± 2 ^a^
4	Prodelphinidin B Isomer	447.1 ± 0.1 ^c^	515.7 ± 0.4 ^b^	715 ± 13 ^a^
5	Gallic acid	153.52 ± 0.09 ^c^	164 ± 3 ^b^	175.9 ± 0.7 ^a^
6	Pedunculagin/Casuariin Isomer	158.8 ± 0.6 ^b^	163.84 ± 0.06 ^b^	175 ± 3 ^a^
7	Pedunculagin/Casuariin Isomer	464.0 ± 0.8 ^c^	475.5 ± 0.5 ^b^	557 ± 2 ^a^
8	Prodelphinidin Dimer Isomer	497 ± 1 ^b^	529 ± 6 ^b^	603 ± 30 ^a^
9	Gallocatechin	4913 ± 47 ^a^	4435 ± 7 ^b^	4098 ± 84 ^c^
10	Vescalagin/castalagin Isomer	157.59 ± 0.01 ^a^	136.6 ± 0.3 ^c^	143 ± 2 ^b^
11	Prodelphinidin Dimer Isomer	1365 ± 7 ^c^	1560 ± 14 ^b^	1739 ± 25 ^a^
12	Uralenneoside	2464 ± 4 ^a^	1911 ± 24 ^b^	1872 ± 81 ^b^
13	Geraniin Isomer	241 ± 1 ^b^	264.9 ± 0.5 ^b^	343 ± 25 ^a^
14	Pedunculagin/Casuariin Isomer	466 ± 3 ^c^	575 ± 16 ^b^	683 ± 20 ^a^
15	Geraniin Isomer	260 ± 3 ^b^	290 ± 7 ^a,b^	356 ± 48 ^a^
16	Procyanidin B Isomer	4262 ± 12 ^c^	4742 ± 15 ^b^	5514 ± 69 ^a^
17	Galloyl(epi)catechin-(epi)gallocatechin	<LOQ	12.60 ± 0.07 ^b^	38 ± 3 ^a^
18	Procyanidin B Isomer	650 ± 3 ^c^	708 ± 11 ^b^	757 ± 23 ^a^
19	Tellimagrandin I Isomer	347 ± 4 ^c^	367.2 ± 0.7 ^b^	397 ± 2 ^a^
20	Pterocarinin A Isomer	569 ± 31 ^b^	617 ± 9 ^b^	679 ± 7 ^a^
21	Pterocarinin A Isomer	316 ± 2 ^c^	360 ± 4 ^b^	376 ± 4 ^a^
22	Stenophyllanin A	853 ± 13 ^c^	1036 ± 50 ^b^	1318 ± 24 ^a^
23	Procyanidin trimer Isomer	781 ± 1 ^a^	706 ± 1 ^c^	738 ± 4 ^b^
24	Catechin	8486 ± 10 ^b^	8957 ± 11 ^a^	6845 ± 24 ^c^
25	Procyanidin tetramer	<LOQ	<LOQ	<LOQ
26	Procyanidin trimer Isomer	89 ± 2 ^c^	108 ± 3 ^b^	128 ± 1 ^a^
27	Guavin A	263 ± 9 ^c^	357 ± 8 ^b^	518 ± 15 ^a^
28	Casuarinin/Casuarictin Isomer	1297 ± 5 ^c^	1568 ± 10 ^b^	2089 ± 11 ^a^
29	Galloyl(epi)catechin-(epi)gallocatechin	61 ± 5 ^c^	135 ± 1 ^b^	211 ± 12 ^a^
30	Procyanidin pentamer	<LOQ	<LOQ	<LOQ
31	Galloyl-(epi)catechin trimer Isomer	<LOQ	<LOQ	<LOQ
32	Gallocatechin	2074 ± 2 ^b^	1526 ± 2 ^c^	2613 ± 55 ^a^
33	Tellimagrandin I Isomer	463 ± 2 ^c^	516 ± 6 ^b^	737 ± 24 ^a^
34	Vescalagin	160 ± 6 ^b^	159 ± 3 ^b^	187 ± 1 ^a^
35	Stenophyllanin A Isomer	355.36 ± 0.07 ^c^	425 ± 13 ^b^	548 ± 20 ^a^
36	Galloyl-(epi)catechin trimer Isomer	<LOQ	<LOQ	<LOQ
37	Myricetin hexoside Isomer	432.10 ± 0.05 ^c^	555 ± 2 ^b^	572 ± 7 ^a^
38	Stachyuranin A	207.40 ± 0.04 ^a^	207 ± 6 ^a^	216 ± 1 ^a^
39	Procyanidin gallate Isomer	533.2 ± 0.07 ^c^	799 ± 3 ^b^	1036 ± 32 ^a^
40	Myricetin hexoside Isomer	213 ± 2 ^c^	288 ± 2 ^b^	307.8 ± 0.8 ^a^
41	Vescalagin/castalagin Isomer	152 ± 3 ^b^	155 ± 2 ^b^	191 ± 3 ^a^
42	Myricetin arabinoside/xylopyranoside Isomer	241 ± 5 ^c^	286 ± 2 ^b^	306 ± 5 ^a^
43	Myricetin arabinoside/xylopyranoside Isomer	608 ± 1 ^c^	839 ± 8 ^b^	946 ± 11 ^a^
44	Procyanidin gallate Isomer	11 ± 1 ^a^	3.7 ± 0.2 ^b^	<LOQ
45	Myricetin arabinoside/xylopyranoside Isomer	688 ± 16 ^c^	816.0 ± 0.5 ^b^	874 ± 9 ^a^
46	Myricetin hexoside Isomer	1186 ± 13 ^a^	1010 ± 3 ^b^	1012 ± 65 ^b^
47	Myricetin hexoside Isomer	200 ± 3 ^b^	208 ± 5 ^b^	224 ± 6 ^a^
48	Myricetin arabinoside/xylopyranoside Isomer	276.0 ± 0.9 ^a,b^	266 ± 3 ^b^	282 ± 8 ^a^
49	Quercetin galloylhexoside Isomer	375.0 ± 0.6 ^b^	380 ± 5 ^b^	438 ± 18 ^a^
50	Ellagic acid deoxyhexoside	700 ± 1 ^a^	702 ± 12 ^a^	733 ± 32 ^a^
51	Quercetin galloylhexoside Isomer	180 ± 2 ^b^	194 ± 7 ^a^	205 ± 1 ^a^
52	Myricetin arabinoside/xylopyranoside Isomer	544.3 ± 0.4 ^b^	525 ± 2 ^b^	588 ± 18 ^a^
53	Morin	2619 ± 4 ^c^	3206 ± 11 ^b^	4474 ± 98 ^a^
54	Myricetin arabinoside/xylopyranoside Isomer	611 ± 4 ^a^	581 ± 6 ^b^	559 ± 3 ^c^
55	Ellagic acid	1229 ± 26 ^c^	1345 ± 34 ^b^	1759.6 ± 0.9 ^a^
56	Hyperin	11305 ± 27 ^c^	11906 ± 57 ^b^	12528 ± 83 ^a^
57	Quercetin glucuronide	<LOQ	<LOQ	<LOQ
58	Isoquercitrin	2254 ± 10 ^b^	2471 ± 16 ^b^	3410 ± 38 ^a^
59	Procyanidin gallate Isomer	<LOQ	7.3 ± 0.3 ^b^	73.3 ± 0.2 ^a^
60	Reynoutrin	2641 ± 11 ^b^	2762 ± 2 ^b^	3210 ± 104 ^a^
61	Guajaverin	8864 ± 8 ^b^	9668 ± 64 ^b^	11813 ± 64 ^a^
62	Guavinoside A	783 ± 5 ^a,b^	770 ± 4 ^b^	793 ± 4 ^a^
63	Avicularin	10353 ± 18 ^a,b^	10173 ± 54 ^b^	11441 ± 63 ^a^
64	Quercitrin	213 ± 2 ^b^	208 ± 2 ^b^	223 ± 1 ^a^
65	Myrciaphenone B	546 ± 6 ^c^	621 ± 1 ^b^	715 ± 20 ^a^
66	Guavinoside C	2069 ± 1 ^b^	1966 ± 21 ^c^	2209 ± 21 ^a^
67	Guavinoside B	872 ± 17 ^c^	1035 ± 23 ^b^	1273 ± 30 ^a^
68	Guavinoside A Isomer	137 ± 1 ^a^	135.1 ± 0.6 ^a^	137 ± 3 ^a^
69	Guavinoside B Isomer	120 ± 2 ^b^	119,6 ± 0.2 ^b^	129 ± 1 ^a^
70	2,6-dihydroxy-3-methyl-4-*O*-(6″-*O*-galloyl-β-d-glucopyranosyl)-benzophenone	1179 ± 12 ^b^	1242 ± 33 ^b^	1365 ± 20 ^a^
71	Guavin B	220.51 ± 0.03 ^b^	230.1 ± 0.7 ^a,b^	241 ± 7 ^a^
72	Quercetin	258 ± 4 ^a^	253 ± 3 ^a^	255 ± 5 ^a^
73	Naringenin	487 ± 3 ^c^	638 ± 24 ^b^	705 ± 6 ^a^
*Positive mode*		Concentration (μg compound/g leaf *d.w.*)		
74	Cyanidin-3-*O*-glucoside	441.28 ± 0.04 ^a^	169.3 ± 0.5 ^b^	29.5 ± 0.2 ^c^

LOQ: limits of quantification; means in the same line with different letter (^a,b,c^) are significantly different (*p* < 0.05).

**Table 2:** Guava leaves’ bioactive compounds related with anti-diabetic properties.

Compound	Assay	Activity	Ref.
Myrciaphenone B	*in vivo*	Inhibition of aldose reductase α-glucosidase	[39]
Casuarictin, tellimagrandin I	*in vitro*	Inhibition of α-glucosidase	[40]
Cyanidin-3-*O*-β-glucoside	*in vitro/in silico*	Inhibition of α-amylase	[43]
Flavonol glycosides	*in vitro*	Inhibition of dipeptidyl-peptidase IV, and α-glucosidase and α-amylase	[30,41]
Geraniin	*in vitro*	Hypoglycemic activity; inhibition of carbohydrate-hydrolysing enzymes (α-glucosidase and α-amylase); effective in preventing advanced glycation end-products (AGEs) formation	[42]
Vescalagin	*in vivo*	Retard AGEs formation	[42]
Gallic acid	*in vitro*	Inhibitory effect on the formation of α-dicarbonyl compounds and protein glycation: inhibitory effects on the production of Amadori products and AGEs	[3,42]
Naringenin	*in vitro*	Anti-glycation activity	[42]
Morin	*in vitro*	Protective activity against glycation	[42]
Quercetin	*in vitro*	Inhibitory effect on protein glycation, on the formation of α-dicarbonyl compounds, and on the production of Amadori products and AGEs	[3,30,42]
Catechin	*in vitro/in vivo/clinical trial*	Inhibitory effect on the formation of α-dicarbonyl compounds and protein glycation: inhibitory effects on the production of Amadori products and AGEs; improvement of postprandial hyperglycaemia	[42,46]
Procyanidin B2	*in vitro/in vivo*	Inhibitory effects on the formation of AGEs	[42]
Casuarinin, casuariin	*in vitro*	Inhibition of insulin-like glucose uptake	[40]
Procyanidin oligomers	*in vitro/in vivo*	Insulinomimetic properties	[44]
Pedunculagin	*in vivo*	Improvement sensitivity of insulin	[45]
Gallocatechin	*clinical trial*	Improvement of postprandial hyperglycaemia	[46]

**Table 3:** Comparison (mean ± SD, *n* = 3) among total phenolic content (TPC) by HPLC-DAD-ESI-QTOF-MS, Trolox Equivalent Antioxidant Capacity (TEAC) and Ferric Reducing Antioxidant Power (FRAP) of *Psidium guajava* L. leaves at different oxidative states.

Oxidation State	TPC (mg/g leaf *d.w.*)	TEAC (mM eq Trolox/mg leaf *d.w.*)	FRAP (mM FeSO_4_/mg leaf *d.w.*)
High	87.91 ± 0.05 ^c^	2.2 ± 0.2 ^c^	3.69 ± 0.03 ^c^
Medium	92.0 ± 0.4 ^b^	2.44 ± 0.05 ^b^	4.20 ± 0.06 ^b^
Low	103 ± 2 ^a^	3.1 ± 0.1 ^a^	5.4 ± 0.1 ^a^

Means in the same column with different letter (^a,b,c^) are significantly different (*p* < 0.05).

## References

[1] Morton J.F. (1987). Fruits of Warm Climates.

[2] Deguchi Y., Miyazaki K. (2010). Anti-hyperglycemic and anti-hyperlipidemic effects of guava leaf extract. Nutr. Metab. (Lond.).

[3] Wu J.-W., Hsieh C.-L., Wang H.-Y., Chen H.-Y. (2009). Inhibitory effects of guava (*Psidium guajava* L.) leaf extracts and its active compounds on the glycation process of protein. Food Chem..

[4] Liu C.-W., Wang Y.-C., Lu H.-C., Chiang W.-D. (2014). Optimization of ultrasound-assisted extraction conditions for total phenols with anti-hyperglycemic activity from *Psidium guajava* leaves. Process Biochem..

[5] Gutiérrez R.M.P., Mitchell S., Solis R.V. (2008). *Psidium guajava*: A review of its traditional uses, phytochemistry and pharmacology. J. Ethnopharmacol..

[6] Bernal J., Mendiola J.A., Ibáñez E., Cifuentes A. (2011). Advanced analysis of nutraceuticals. J. Pharm. Biomed. Anal..

[7] Salazar D.M., Melgarejo P., Martínez R., Martínez J.J., Hernández F., Burguera M. (2006). Phenological stages of the guava tree (*Psidium guajava* L.). Sci. Hortic. (Amsterdam).

[8] Hao W. (2008). Freezing Tolerance and Cold Acclimation in Guava (*Psidium guajava* L.). Graduate Theses and Dissertations.

[9] Vargas-Alvarez D., Soto-Hernández M., González-Hernández V.A., Engleman E.M., Martínez-Garza Á. (2006). Kinetics of accumulation and distribution of flavonoids in guava (*Psidium guajava* L.). Agrociencia.

[10] Kajdžanoska M., Gjamovski V., Stefova M. (2010). HPLC-DAD-ESI-MSn identification of phenolic compounds in cultivated strawberries from macedonia. Maced. J. Chem. Chem. Eng..

[11] Heng M.Y., Tan S.N., Yong J.W.H., Ong E.S. (2013). Emerging green technologies for the chemical standardization of botanicals and herbal preparations. TrAC Trends Anal. Chem..

[12] Nantitanon W., Yotsawimonwat S., Okonogi S. (2010). Factors influencing antioxidant activities and total phenolic content of guava leaf extract. LWT-Food Sci. Technol..

[13] Venkatachalam R.N., Singh K., Marar T. (2012). Phytochemical screening *in vitro* antioxidant activity of *Psidium guajava*. Free Radic. Antiox..

[14] Seo J., Lee S., Elam M.L., Johnson S.A., Kang J., Arjmandi B.H. (2014). Study to find the best extraction solvent for use with guava leaves (*Psidium guajava* L.) for high antioxidant efficacy. Food Sci. Nutr..

[15] Mailoa M.N., Mahendradatta M., Laga A., Djide N. (2013). Tannin extract of guava leaves (*Psidium guajava* L) variation with concentration organic solvents. Int. J. Sci. Technol. Res..

[16] Chang C.-H., Hsieh C.-L., Wang H.-E., Peng C.-C., Chyau C.-C., Peng R.Y. (2013). Unique bioactive polyphenolic profile of guava (*Psidium guajava*) budding leaf tea is related to plant biochemistry of budding leaves in early dawn. J. Sci. Food Agric..

[17] Díaz-de-Cerio E., Verardo V., Gómez-Caravaca A.M., Fernández-Gutiérrez A., Segura-Carretero A. (2015). Determination of polar compounds in guava leaves infusions and ultrasound aqueous extract by HPLC-ESI-MS. J. Chem..

[18] Díaz-de-Cerio E., Gómez-Caravaca A.M., Verardo V., Fernández-Gutiérrez A., Segura-Carretero A. (2016). Determination of guava (*Psidium guajava* L.) leaf phenolic compounds using HPLC-DAD-QTOF-MS. J. Funct. Food.

[19] Jang M., Jeong S.-W., Cho S.K., Yang H.J., Yoon D.-S., Kim J.-C., Park K.-H. (2014). Improvement in the anti-inflammatory activity of guava (*Psidium guajava* L.) leaf extracts through optimization of extraction conditions. J. Funct. Foods.

[20] Zhu Y., Liu Y., Zhan Y., Liu L., Xu Y., Xu T., Liu T. (2013). Preparative isolation and purification of five flavonoid glycosides and one benzophenone galloyl glycoside from *Psidium guajava* by high-speed counter-current chromatography (HSCCC). Molecules.

[21] Qu C., Fu F., Lu K., Zhang K., Wang R., Xu X., Wang M., Lu J., Wan H., Zhanglin T. (2013). Differential accumulation of phenolic compounds and expression of related genes in black- and yellow-seeded *Brassica napus*. J. Exp. Bot..

[22] Holton T., Cornish E. (1995). Genetics and biochemistry of anthocyanin biosynthesis. Plant Cell.

[23] Coley P.D., Barone J.A. (1996). Herbivory and plant defenses in tropical forests. Annu. Rev. Ecol. Syst..

[24] Soltani N., Wagner D. (2011). Prevention of Diabetes Complications. Type 1 Diabetes Complications.

[25] Chao H., Wu P., Lo D., Wu W., Wu M. (2013). Effect of guava (*Psidium guajava* Linn.) fruit water extract on lipid peroxidation and serum lipid profiles of streptozotocin-nicotinamide induced diabetic rats. Afr. J. Pharm. Pharmacol..

[26] Rai P.K., Mehta S., Watal G. (2010). Hypolipidaemic & hepatoprotective effects of *Psidium guajava* raw fruit peel in experimental diabetes. Indian J. Med. Res..

[27] Farinazzi-Machado F.M.V., Landgraf Guiguer É., Barbalho S.M., da Silva Soares de Souza M., Cincotto dos Santos Bueno P., Gregório Mendes C., Cressoni Araújo A., Rezende Teixeira Rodrigues A., de Lara Lima L.M., Sanches Marim N. (2012). Effects of *Psidium guajava* on the metabolic profile of Wister rats. J. Med. Plants Res..

[28] Mukhtar H.M., Ansari S.H., Bhat Z.A., Naved T., Singh P. (2006). Antidiabetic activity of an ethanol extract obtained from the stem bark of *Psidium guajava* (Myrtaceae). Die Pharm. Int. J. Pharm. Sci..

[29] Singh R., Kaur N., Kishore L., Gupta G.K. (2013). Management of diabetic complications: A chemical constituents based approach. J. Ethnopharmacol..

[30] Wang H., Du Y.-J., Song H.-C. (2010). α-Glucosidase and α-amylase inhibitory activities of guava leaves. Food Chem..

[31] Jiménez-Escrig A., Rincón M., Pulido R., Saura-Calixto F. (2001). Guava fruit (*Psidium guajava* L.) as a new source of antioxidant dietary fiber. J. Agric. Food Chem..

[32] Ribeiro da Silva L.M., Teixeira de Figueiredo E.A., Silva Ricardo N.M.P., Pinto Vieira I.G., Wilane de Figueiredo R., Brasil I.M., Gomes C.L. (2014). Quantification of bioactive compounds in pulps and by-products of tropical fruits from Brazil. Food Chem..

[33] Gamal F.M., Samira S.M., Fakhriya S.T. (2011). Antioxidant, antimicrobial and anticarcinogenic properties of Egyptian guava seeds extracts. Nat. Sci..

[34] Aminu M., Bello M.S., Abbas O., Aliyu M. (2012). Comparative *in vitro* antioxidant studies of ethanolic extracts of *Psidium guajava* stem bark and *Telfairia occidentalis* leaf. Int. J. Mod. Biochem..

[35] Oboh G., Akinyemi A.J., Ademiluyi A.O. (2012). Inhibition of α-amylase and α-glucosidase activities by ethanolic extract of *Telfairia occidentalis* (fluted pumpkin) leaf. Asian Pac. J. Trop. Biomed..

[36] Stankovic M.S., Niciforovic N., Mihailovic V., Topuzovic M., Solujic S. (2012). Antioxidant activity, total phenolic content and flavonoid concentrations of different plant parts of *Teucrium polium* L. subsp. polium. Acta Soc. Bot. Pol..

[37] Yang C.-H., Li R.-X., Chuang L.-Y. (2012). Antioxidant activity of various parts of *Cinnamomum cassia* extracted with different extraction methods. Molecules.

[38] Beato V.M., Orgaz F., Mansilla F., Montaño A. (2011). Changes in phenolic compounds in garlic (*Allium sativum* L.) owing to the cultivar and location of growth. Plant Foods Hum. Nutr..

[39] Yoshikawa M., Shimada H., Nishida N., Li Y., Toguchida I., Yamahara J., Matsuda H. (1998). Antidiabetic principles of natural medicines. II. Aldose reductase and α-glucosidase inhibitors from Brazilian natural medicine, the leaves of Myrcia multiflora DC. (Myrtaceae): Structures of myrciacitrins I and II and myrciaphenones A and B. Chem. Pharm. Bull. (Tokyo).

[40] Yoshida T., Amakura Y., Yoshimura M. (2010). Structural features and biological properties of ellagitannins in some plant families of the order myrtales. Int. J. Mol. Sci..

[41] Eidenberger T., Selg M., Krennhuber K. (2013). Inhibition of dipeptidyl peptidase activity by flavonol glycosides of guava (*Psidium guajava* L.): A key to the beneficial effects of guava in type II diabetes mellitus. Fitoterapia.

[42] Chinchansure A.A., Korwar A.M., Kulkarni M.J., Joshi S.P. (2015). Recent development of plant products with anti-glycation activity: A review. RSC Adv..

[43] Sui X., Zhang Y., Zhou W. (2016). *In vitro* and *in silico* studies of the inhibition activity of anthocyanins against porcine pancreatic α-amylase. J. Funct. Foods.

[44] Pinent M., Blay M., Bladé M.C., Salvadó M.J., Arola L., Ardévol A. (2004). Grape seed-derived procyanidins have an antihyperglycemic effect in streptozotocin-induced diabetic rats and insulinomimetic activity in insulin-sensitive cell lines. Endocrinology.

[45] Patel D.K., Prasad S.K., Kumar R., Hemalatha S. (2012). An overview on antidiabetic medicinal plants having insulin mimetic property. Asian Pac. J. Trop. Biomed..

[46] Takahashi M., Miyashita M., Suzuki K., Bae S.-R., Kim H.-K., Wakisaka T., Matsui Y., Takeshita M., Yasunaga K. (2014). Acute ingestion of catechin-rich green tea improves postprandial glucose status and increases serum thioredoxin concentrations in postmenopausal women. Br. J. Nutr..

[47] Li S., Li S.-K., Gan R.-Y., Song F.-L., Kuang L., Li H.-B. (2013). Antioxidant capacities and total phenolic contents of infusions from 223 medicinal plants. Ind. Crop Prod..

[48] Tachakittirungrod S., Okonogi S., Chowwanapoonpohn S. (2007). Study on antioxidant activity of certain plants in Thailand: Mechanism of antioxidant action of guava leaf extract. Food Chem..

[49] Nantitanon W. (2012). Comparison of antioxidant activity of compounds isolated from guava leaves and a stability study of the most active compound. Drug Discov. Ther..

[50] Thaipong K., Boonprakob U., Crosby K., Cisneros-Zevallos L., Hawkins Byrne D. (2006). Comparison of ABTS, DPPH, FRAP, and ORAC assays for estimating antioxidant activity from guava fruit extracts. J. Food Compos. Anal..

[51] Dudonne S., Vitrac X., Coutiere P., Woillez M., Merillon J.-M. (2009). Comparative study of antioxidant properties and total phenolic content of 30 plant extracts of industrial interest using DPPH, ABTS, FRAP, SOD, and ORAC assays. J. Agric. Food Chem..

[52] Laporta O., Perezfons L., Mallavia R., Caturla N., Micol V. (2007). Isolation, characterization and antioxidant capacity assessment of the bioactive compounds derived from *Hypoxis rooperi* corm extract (African potato). Food Chem..

[53] Benzie I.F., Strain J.J. (1996). The ferric reducing ability of plasma (FRAP) as a measure of “antioxidant power”: The FRAP assay. Anal. Biochem..

[54] Gómez-Caravaca A.M., Verardo V., Toselli M., Segura-Carretero A., Fernández-Gutiérrez A., Caboni M.F. (2013). Determination of the major phenolic compounds in pomegranate juices by HPLC-DAD-ESI-MS. J. Agric. Food Chem..

